# Pentraxin 3 deficiency exacerbates neutrophilic inflammation and airway hyperresponsiveness in type 2-low asthma

**DOI:** 10.3389/falgy.2026.1731295

**Published:** 2026-02-05

**Authors:** Sina Taefehshokr, Lianyu Shan, Mojdeh Matloubi, Sujata Basu, A. Halayko, Abdelilah S. Gounni

**Affiliations:** 1 Department of Immunology, Rady Faculty of Health Sciences, Max Rady College of Medicine, University of Manitoba, Winnipeg, MB, Canada; 2 Department of Physiology and Pathophysiology, Rady Faculty of Health Sciences, Max Rady College of Medicine, University of Manitoba, Winnipeg, MB, Canada

**Keywords:** airway inflammation, airway hyperresponsiveness, neutrophil, PTX3, type 2-low asthma

## Abstract

**Background:**

Type 2-low asthma is a severe, steroid-resistant phenotype characterized by neutrophilic inflammation and limited treatment options. PTX3, an acute-phase protein involved in innate immunity, has been linked to inflammatory diseases; but its role in type 2-low asthma remains unclear.

**Methods:**

A chronic HDM + c-di-GMP murine model was used to mimic type 2-low asthma. PTX3^−/−^ and WT mice were assessed for inflammation, cytokine profiles, antibody responses, and lung function. AHR was measured using FlexiVent. BALF inflammatory cells were analyzed by cytospin and flow cytometry. Cytokines were quantified using mesoscale assay, and serum immunoglobulins by ELISA.

**Results:**

In mice, the type 2-low model exhibited increased systemic and airway PTX3 levels. PTX3^−/−^ mice exposed to the type 2-low protocol developed significantly greater airway inflammation, with higher total BALF cell counts and a 2-fold increase in neutrophils, but no change in eosinophils. PTX3 deficiency led to increased total and HDM-specific IgE levels. BALF cytokine analysis revealed elevated IL-17A in PTX3^−/−^ mice, while IL-4, IL-5, and IL-13 remained unchanged. PTX3^−/−^ mice also exhibited significantly higher AHR parameters.

**Conclusions:**

PTX3 absence enhances neutrophilic inflammation, IL-17A production, IgE responses, and AHR, highlighting PTX3 as a potential biomarker and therapeutic target in type 2- low asthma.

## Introduction

Asthma is a chronic inflammatory disease of the airways characterized by reversible airflow obstruction, bronchial hyperresponsiveness, and airway remodeling. Globally, asthma affects hundreds of millions of people and contributes substantially to morbidity and healthcare burden (1–3).

Asthma is now understood to encompass multiple phenotypes and endotypes with distinct immunopathology. Type 2-high asthma is typically driven by Th2 cytokines (IL-4, IL-5, IL-13) and eosinophilic inflammation, and patients usually respond well to corticosteroids and Th2-targeted therapies (4). In contrast, type 2-low asthma is often characterized by neutrophil-dominated or paucigranulocytic airway inflammation and a relative lack of Th2 cytokine signatures (5). This phenotype is associated with more severe, refractory disease that is less responsive to inhaled steroids. Indeed, clinical studies have identified “neutrophilic asthma” as a distinct inflammatory subtype linked to frequent exacerbations and corticosteroid insensitivity (6, 7). There are currently no effective targeted therapies for type 2-low asthma, highlighting an urgent need to identify key inflammatory mediators driving this subtype and to develop novel interventions.

PTX3 is an evolutionarily conserved 42 kDa protein belonging to the long pentraxin family (8). Unlike the short pentraxins (e.g., C-reactive protein), PTX3 is produced at sites of inflammation by a variety of cells (including dendritic cells, macrophages, endothelial and epithelial cells) in response to pro-inflammatory signals (e.g., IL-1β and TNF) (9–11). PTX3 acts as a soluble pattern recognition receptor that can bind microbial moieties and molecules like complement components, thereby orchestrating innate immune responses. Through these interactions, PTX3 facilitates pathogen recognition, modulates leukocyte recruitment, and promotes the resolution of inflammation (12). Importantly, PTX3 has known regulatory effects on neutrophils; it can bind adhesion molecules such as P-selectin on endothelial cells, dampening excessive neutrophil transmigration into tissues (13). PTX3 also influences inflammatory cytokine milieus; for example, it can modulate IL-1β and IL-17-driven pathways that are central to neutrophilic inflammation (14). Given these functions, PTX3 is emerging as an immunoregulatory molecule in conditions characterized by neutrophil-dominant inflammation. PTX3 expression is elevated in the bronchial biopsies of asthmatic individuals (15). However, the clinical studies to date have not distinguished between asthma endotypes, and it remains unclear whether PTX3 contributes to the pathogenesis of type 2-low asthma. In a previous murine study of allergic asthma, PTX3 deficiency was found to aggravate airway inflammation with a skewing toward a Th17/neutrophilic response (16). This suggests that PTX3 may normally act to constrain certain inflammatory pathways in the lungs.

We hypothesized that PTX3 plays a protective, anti-inflammatory role in neutrophilic asthma, and that lack of PTX3 would exacerbate airway neutrophilia and hyperresponsiveness in a type 2-low context. To investigate this, employed a previously described chronic house dust mite–driven murine model incorporating c-di-GMP, a potent inducer of neutrophil- and IL-17–mediated immunity, to recapitulate key features of type 2-low asthma (17). Using PTX3 KO mice in this model, we assessed the impact of PTX3 deficiency on airway inflammation, immunoglobulin production, cytokine profiles, and airway hyperresponsiveness. Our study thus evaluates PTX3 as a potential immunoregulator in steroid-resistant, neutrophilic asthma, with implications for future therapies targeting this pathway.

## Materials and methods

### Animals

Female and male PTX3 KO and WT mice on a 129SvEv/Bl/6 background, aged 5–8 weeks, were used in the study. PTX3 knockout mice were generated via homologous recombination to delete exons 1 and 2 of the ptx3 gene, including the start codon and signal peptide, resulting in a null mutation (18). Founder mice were provided by Dr. M. Matzuk (Baylor College of Medicine) and bred at the University of Manitoba animal facility. All animal experiments adhered to Canadian Council on Animal Care guidelines and were approved by the University of Manitoba Animal Ethics Board (Protocol #23-042/1). To safeguard animal welfare, predefined humane endpoints were established before the study commenced. Animals were observed at least twice daily for any signs of illness or distress, such as hunching, difficulty breathing, decreased movement, lethargy, raised fur, poor grooming, or a body weight loss greater than 15% from baseline. If any of these indicators were present, the animal was promptly euthanized. Euthanasia was carried out under deep anesthesia with isoflurane, followed by cervical dislocation to confirm death, in accordance with institutional guidelines. The interval between meeting endpoint criteria and euthanasia was kept under 12 h. No animals died unexpectedly before reaching the predefined criteria. All personnel involved in animal care were trained in identifying humane endpoints and performing euthanasia. As the study did not involve surgery and only induced temporary immune responses, analgesics were not necessary. All possible measures were taken to reduce animal discomfort and distress. For experimental rigor, Mice were allocated to experimental groups to ensure comparable age and sex distribution across groups. Group allocation was performed prior to the initiation of HDM or HDM + c-di-GMP exposures. Investigators performing BALF differential cell counts were blinded to genotype and treatment group. Lung mechanics measurements (FlexiVent) and cytokine quantification were performed using automated systems with predefined acquisition and analysis parameters. Flow cytometry data were analyzed using standardized gating strategies applied uniformly across samples; sample identities were coded during analysis to minimize bias.

### HDM sensitization and asthma models

Mice were assigned to different experimental asthma models. In the type 2-high model, mice were sensitized intranasally with 25 µg of HDM extract (lot 259,585; Greer Laboratories, Lenoir, NC, protein concentration: 6.08 mg protein/vial, LPS concentration: 115 EU/mg) on days 1, 3, and 5. Intranasal challenges with the same dose were performed on days 11–13, 18–20, and 25–28. Mice were euthanized on day 28 for sample collection. For the type 2-low model, mice were sensitized with 25 µg HDM plus 5 µg c-di-GMP intranasally on days 1, 3, and 5. Challenges were performed on days 11–13 and 18–20 with 25 µg HDM plus 0.5 µg c-di-GMP, followed by HDM-only challenges on days 25–28. Mice were sacrificed on day 28 for tissue collection.

### Lung function and airway hyperresponsiveness

Airway mechanics were assessed using the FlexiVent system (SCIREQ, Montreal, Canada) after two weeks of treatment. Mice were anesthetized intraperitoneally with pentobarbital (70–90 mg/kg), tracheostomized, and ventilated with a tidal volume of 10 mL/kg at 150 breaths/min and 3 cmH₂O PEEP. Methacholine (3–50 mg/mL) was administered via nebulization. Measurements were obtained using the low-frequency forced oscillation technique (2.5 Hz) and fitted to the constant-phase model to derive Newtonian resistance (Rn), total lung resistance (Rrs), tissue damping (G), and tissue elastance (H). Baseline values were obtained using saline. A coefficient of determination (COD) threshold of 0.9 was applied to exclude unreliable measurements. Measurements failing to meet the COD ≥ 0.9 criterion accounted for fewer than 5% of total measurements and did not result in the exclusion of any animals from the analysis.

### BALF collection

Following euthanasia, BALF was collected via tracheal cannulation and lavage with 1 mL of sterile PBS containing 0.5 mM EDTA. Two washes were performed, and fluids were pooled, centrifuged at 1,000–1,200 rpm (5–10 min, 4 °C). Supernatants were stored at −80 °C for cytokine analysis; cell pellets were resuspended in PBS for cytospin and counting. Total cells were counted using trypan blue exclusion and a hemocytometer. Cytospin slides (1 × 10^5^ cells/slide) were stained using PROTOCOL Hema 3 solutions and evaluated under 40× magnification. Differential cell counts (≥200 cells) were performed in a blinded fashion by two independent observers.

### Single cell suspension preparation

Lung tissues were minced and digested in RPMI 1,640 containing 1 mg/mL collagenase IV and 0.5 mg/mL DNase. Tissue was filtered through a 70 µm cell strainer, red blood cells were lysed, and cells were resuspended in complete RPMI. Viability was assessed using trypan blue exclusion, and cells were counted using a hemocytometer under 40× magnification.

### Flow cytometry

Lung single-cell suspensions (2 × 10^6^ cells) were stained using standard protocols. After Fc blocking, cells were incubated with anti-mouse antibodies, including CD4 (APC-Cy7, BD Pharmingen, Clone: GK 1.5, Lot#: B381129), CD11c (eFluor 450, eBioscience, Clone: N418, Lot#: 4,300,055), SiglecF (PE, BioLegend, Clone: S17007L, Lot#: B357623), CD3e (PE-Cy7, BioLegend, Clone: 17A2, Lot#: B356288), CD45 (APC-Cy7, BioLegend, Clone: 13/2.3, Lot#: B362428), CD11b (PE-Cy7, BioLegend, Clone: M1/70, Lot#: B390649), Gr-1 (Ly6G) (FITC, eBioscience, Clone: RB6-8C5, Lot#: 4,322,602), B220 (APC, BD Pharmingen, Clone: RA3-6B2, Lot#: 7,153,546), and MHC II (PB, BioLegend, Clone: M5/114.152, Lot#: B360614). Zombie Aqua™ viability dye and 4% paraformaldehyde were used for live/dead staining and fixation. Data were acquired using a BD CytoFLEX LX flow cytometer (Beckman Coulter) and analyzed in FlowJo v10.81. At least 100,000 events were collected per sample.

### Cytokines quantification

BALF cytokine concentrations (TNF, IL-6, KC/GRO, IL-4, IL-5, IL-13, IL-17A, IFN-γ) were measured using the MSD U-PLEX system (Cat# K15069M-1), following the manufacturer's instructions. Plates were read on a MESO QuickPlex SQ120 instrument, and data were analyzed using GraphPad Prism.

### Serum collection and immunoglobulin ELISA

Blood was collected via cardiac puncture, and serum was separated by centrifugation (10,000 × g, 5 min, 4 °C). ELISAs were conducted on 96-well plates coated with HDM (for antigen-specific detection) or capture antibodies (for total IgE, IgG1, IgG2a, IgG2b). Samples were blocked, incubated with serially diluted sera, and detected with biotinylated secondary antibodies. After substrate reaction, absorbance was measured at 450 nm (SpectraMax 190), and data were analyzed using SoftMax Pro v5.4.1.

### Statistical analysis

All statistical analyses were performed using GraphPad Prism 10. Data are presented as mean ± SEM. Prior to parametric testing, data distributions were visually inspected and assessed for normality within Prism. Group comparisons were analyzed using one-way ANOVA for single-endpoint comparisons or two-way repeated-measures ANOVA for methacholine dose-response curves, with Bonferroni *post hoc* correction for multiple comparisons within each figure.

Repeated-measures analyses accounted for within-mouse responses across increasing methacholine doses. No missing data points were present for the reported analyses. Primary endpoints included airway hyperresponsiveness parameters and BALF inflammatory cell counts; cytokine and immunoglobulin measurements were considered secondary outcomes. A *P*-value < 0.05 was considered statistically significant.

## Results

### PTX3 expression is elevated in severe asthma and in a murine model of type 2-low asthma with neutrophilic inflammation

**Figure 1:**
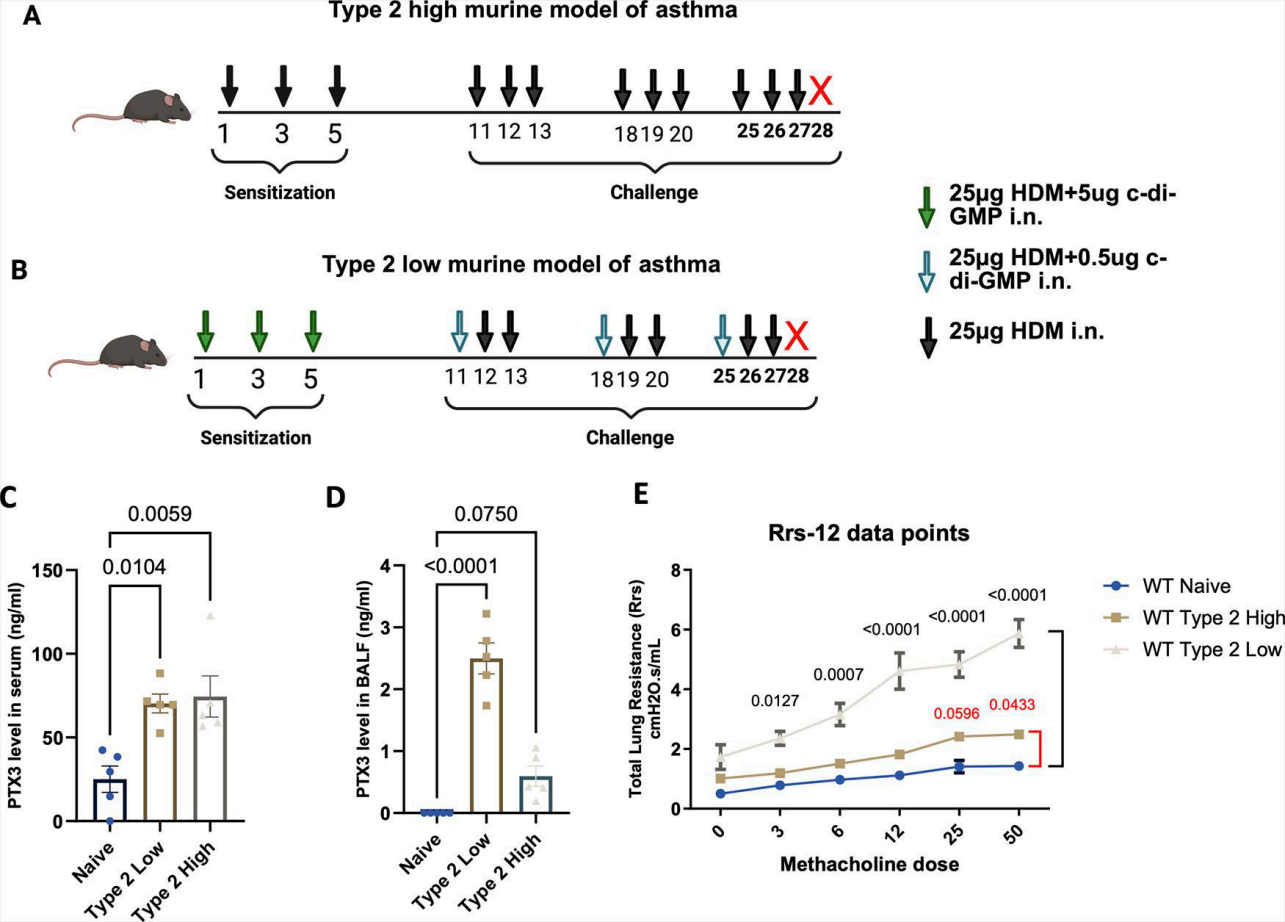
Experimental design and PTX3 measurements in murine asthma models alongside with lung function. **(A,B)** Schematic of murine asthma models. **(A)** Type 2-high model: mice were sensitized and challenged intranasally with HDM alone. **(B)** Type 2-low model: mice received HDM plus c-di-GMP during both sensitization and challenge phases. **(C,D)** PTX3 levels measured by ELISA in murine serum **(C)** and BALF **(D)** from naïve, type 2-high, and type 2-low groups. **(E)** Total lung resistance (Rrs) using the FlexiVent system. Each dot represents one individual biological replicate (mouse). Statistical analysis was performed using one-way ANOVA. *P* values: * < 0.05, ** < 0.01, *** < 0.001, **** < 0.0001.

To model distinct inflammatory endotypes of asthma, we employed two chronic house dust mite–driven murine models previously established by our laboratory (Figures 1A,B) (19). In the type 2-high model (Figure 1A), mice were sensitized and challenged intranasally with HDM extract alone, leading to eosinophil-predominant airway inflammation. In contrast, the type 2-low model (Figure 1B) incorporated the same house dust mite protocol but included cyclic-di-GMP, a bacterial second messenger known to activate innate immunity and promote interleukin-17–mediated neutrophilic inflammation.

We assessed PTX3 levels in the murine models. Serum PTX3 concentrations were significantly elevated in both type 2-high and type 2-low mice compared to naïve controls (Figure 1C). However, when PTX3 was measured in the bronchoalveolar lavage fluid, only the type 2-low group demonstrated a significant increase relative to naïve mice, whereas the type 2-high group showed only a modest, non-significant change (Figure 1D). This suggests that airway-localized PTX3 production is selectively enhanced in the type 2-low model. Lung function testing further showed greater airway hyperresponsiveness (Total lung resistance) in the type 2-low group (Figure 1E).

To validate the inflammatory profiles, we quantified airway neutrophils and eosinophils. As expected, the type 2-low model showed significantly increased BALF neutrophils compared to naïve and type 2-high groups (Supplementary Figure 1SA), while eosinophils were predominantly elevated in the type 2-high group (Supplementary Figure 1SB). Importantly, local PTX3 expression was markedly enhanced in the type 2-low model, supporting its relevance to neutrophilic asthma.

### PTX3 deficiency exacerbates airway neutrophilia in the type 2-low asthma model

**Figure 2:**
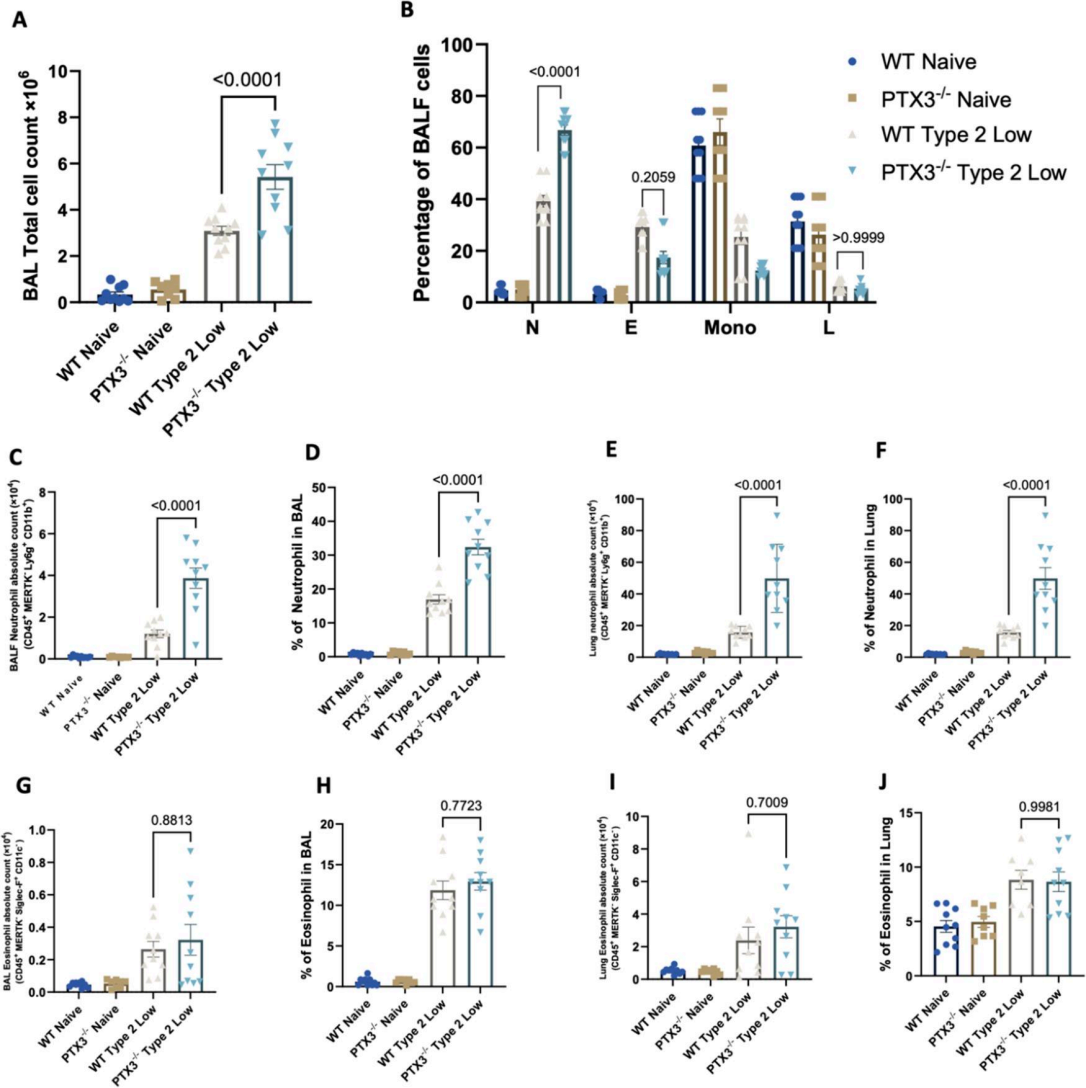
PTX3 deficiency enhances neutrophilic but not eosinophilic inflammation in the type 2-low asthma model. (A) Total BALF cell count. (B) Percentage of neutrophils (N), eosinophils (E), mononuclear cells (Mono), and lymphocytes (L) in BALF determined from cytospin slides by manual differential counting. (C,D) Absolute number (C) and percentage (D) of neutrophils in BALF determined by flow cytometry. (E,F) Absolute number (E) and percentage (F) of neutrophils in lung tissue. (G,H) Absolute number (G) and percentage (H) of eosinophils in BALF. (I,J) Absolute number (I) and percentage (J) of eosinophils in lung tissue. Each dot represents one individual biological replicate (mouse). Statistical analysis was performed using one-way ANOVA. *P* values are reported on the figure.

To determine the impact of PTX3 on airway inflammation, WT and PTX3^−/−^ mice were exposed to the type 2-low asthma protocol. BALF total cell counts were significantly increased in PTX3^−/−^ mice compared to all other groups, including WT type 2-low mice (Figure 2A).

To characterize the composition of the inflammatory cells, cytospin slides of BALF were prepared and stained for manual differential cell counting. Analysis of cell differentials showed that neutrophils were the predominant cell type elevated in PTX3^−/−^ type 2-low mice (Figure 2B). Eosinophils remained low across all groups, with a slight increase in the WT type 2-low group that was not statistically significant in PTX3^−/−^ mice.

Flow cytometric analysis confirmed a marked increase in the absolute number (Figure 2C) and percentage (Figure 2D) of neutrophils in BALF from PTX3^−/−^ type 2-low mice compared to WT controls. Similarly, lung tissue analysis showed significantly elevated neutrophil counts (Figure 2E) and frequency (Figure 2F) in PTX3^−/−^ mice. In contrast, eosinophil counts and percentages in both BALF (Figures 2G,H) and lung tissue (Supplementary Figures 2I,J) were not significantly different between PTX3^−/−^ and WT mice in either naïve or type 2-low groups. These findings indicate that the absence of PTX3 selectively is associated with amplified neutrophilic airway inflammation in the type 2-low asthma context.

### PTX3 deficiency enhances IgE responses in the type 2-low asthma model

HDM exposure is known to trigger systemic immunoglobulin production, contributing to allergic airway inflammation (20, 21). To assess the impact of PTX3 deficiency on the humoral immune response in the type 2-low asthma model, total and HDM-specific immunoglobulin levels were measured in serum from WT and PTX3^−/−^ mice.

**Figure 3:**
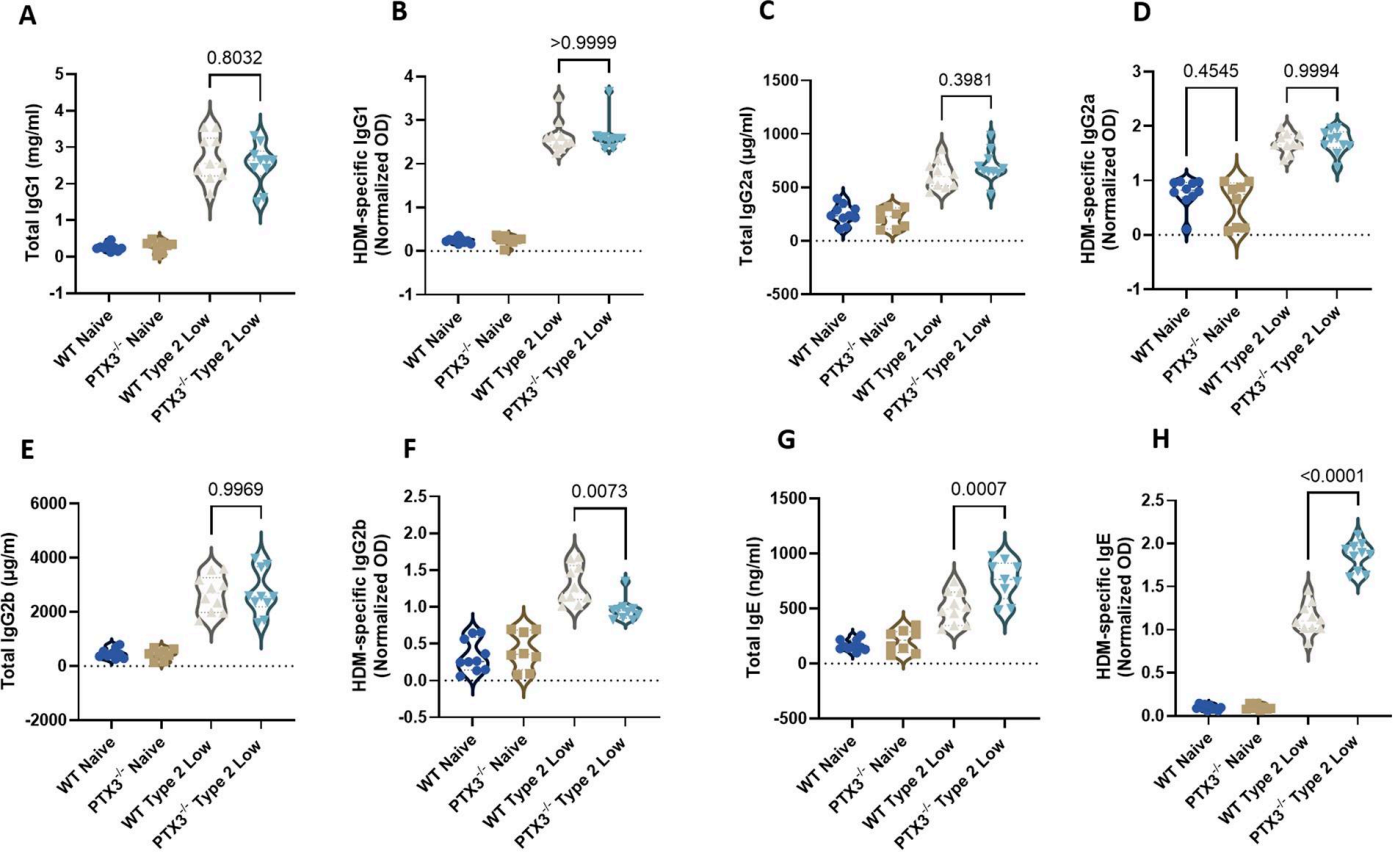
PTX3 deficiency enhances IgE responses in the type 2-low asthma model. **(A,B)** Total IgG1 **(A)** and HDM-specific IgG1 **(B)** measured in serum. **(C,D)** Total IgG2a **(C)** and HDM-specific IgG2a **(D)**. **(E,F)** Total IgG2b **(E)** and HDM-specific IgG2b **(F)**. **(G,H)** Total IgE **(G)** and HDM-specific IgE **(H)**. HDM-specific immunoglobulin levels were determined by ELISA and expressed as normalized optical density (OD). Each dot represents one individual biological replicate (mouse). Statistical analysis was performed using one-way ANOVA. *P* values are reported on the figure.

There were no significant differences between WT and PTX3^−/−^ type 2-low mice in total IgG1 (Figure 3A), HDM-specific IgG1 (Figure 3B), total IgG2a (Figure 3C), or HDM-specific IgG2a (Figure 3D). Similarly, total IgG2b levels were comparable between groups (Figure 3E), although HDM-specific IgG2b was significantly decreased in PTX3^−/−^ mice compared to WT controls (Figure 3F).

Importantly, PTX3^−/−^ mice showed a significant elevation in both total IgE (Figure 3G) and HDM-specific IgE (Figure 3H) relative to WT type 2-low animals, indicating an exaggerated IgE-mediated humoral response in the absence of PTX3.

These findings suggest that PTX3 modulates antigen-specific antibody responses in type 2-low asthma, particularly by restraining IgE production.

### PTX3^−/−^ mice exhibit increased interleukin-17A levels in the type 2-low asthma model

HDM exposure is known to elevate cytokine levels in BALF, contributing to airway inflammation. To assess how PTX3 deficiency affects the local inflammatory milieu, we measured cytokine levels in BALF using the MesoScale Discovery platform.

**Figure 4:**
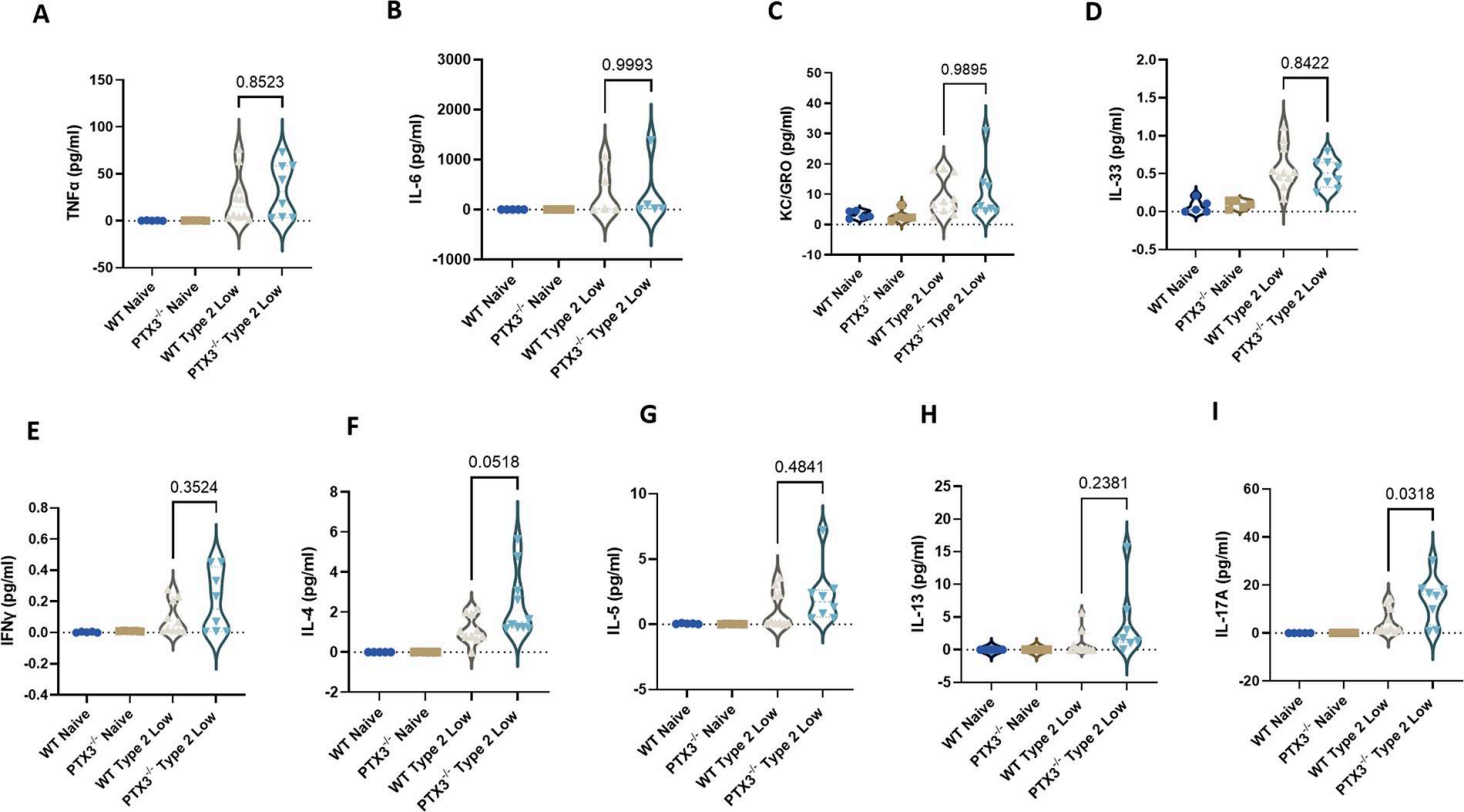
Cytokine levels in BALF from WT and PTX3^−/−^ mice in the type 2-low asthma model. **(A–D)** TNF, IL-6, KC/GRO, and IL-33 levels; **(E–H)** IFN-γ, IL-4, IL-5, and IL-13 levels; **(I)** IL-17A levels. Cytokines were quantified in BALF using the MesoScale Discovery U-PLEX platform and analyzed within the manufacturer-reported lower limits of detection for each analyte (TNF: 1.3 pg/mL; IL-6: 4.8 pg/mL; KC/GRO: 0.43 pg/mL; IL-33: 2.2 pg/mL; IFN-γ: 0.16 pg/mL; IL-4: 0.56 pg/mL; IL-5: 0.63 pg/mL; IL-13: 2.7 pg/mL; IL-17A: 0.30 pg/mL). Each dot represents one individual biological replicate (mouse). Statistical analysis was performed using one-way ANOVA. *P* values are reported on the figure.

There were no significant differences in TNF, IL-6, KC/GRO, or IL-33 between PTX3^−/−^ and WT type 2-low mice (Figures 4A–D). Similarly, levels of Th1 and Th2 cytokines, IFN-γ, IL-4, IL-5, and IL-13 remained comparable between the groups (Figures 4E–H).

Notably, PTX3^−/−^ type 2-low mice showed a significant increase in IL-17A levels compared to WT type 2-low controls (Figure 4I), indicating a shift toward a T helper 17 skewed response in the absence of PTX3.

These findings suggest that compared to WT, PTX3 deficiency does not alter the levels of classical type 2 or pro-inflammatory cytokines in the BALF, but is associated with elevated IL-17A, which may contribute to the heightened neutrophilic inflammation observed in PTX3^−/−^ mice.

### PTX3^−/−^ mice exhibit elevated AHR in the type 2-low asthma model

To determine whether PTX3 deficiency affects lung function, we assessed AHR using the FlexiVent system in response to increasing doses of methacholine.

**Figure 5:**
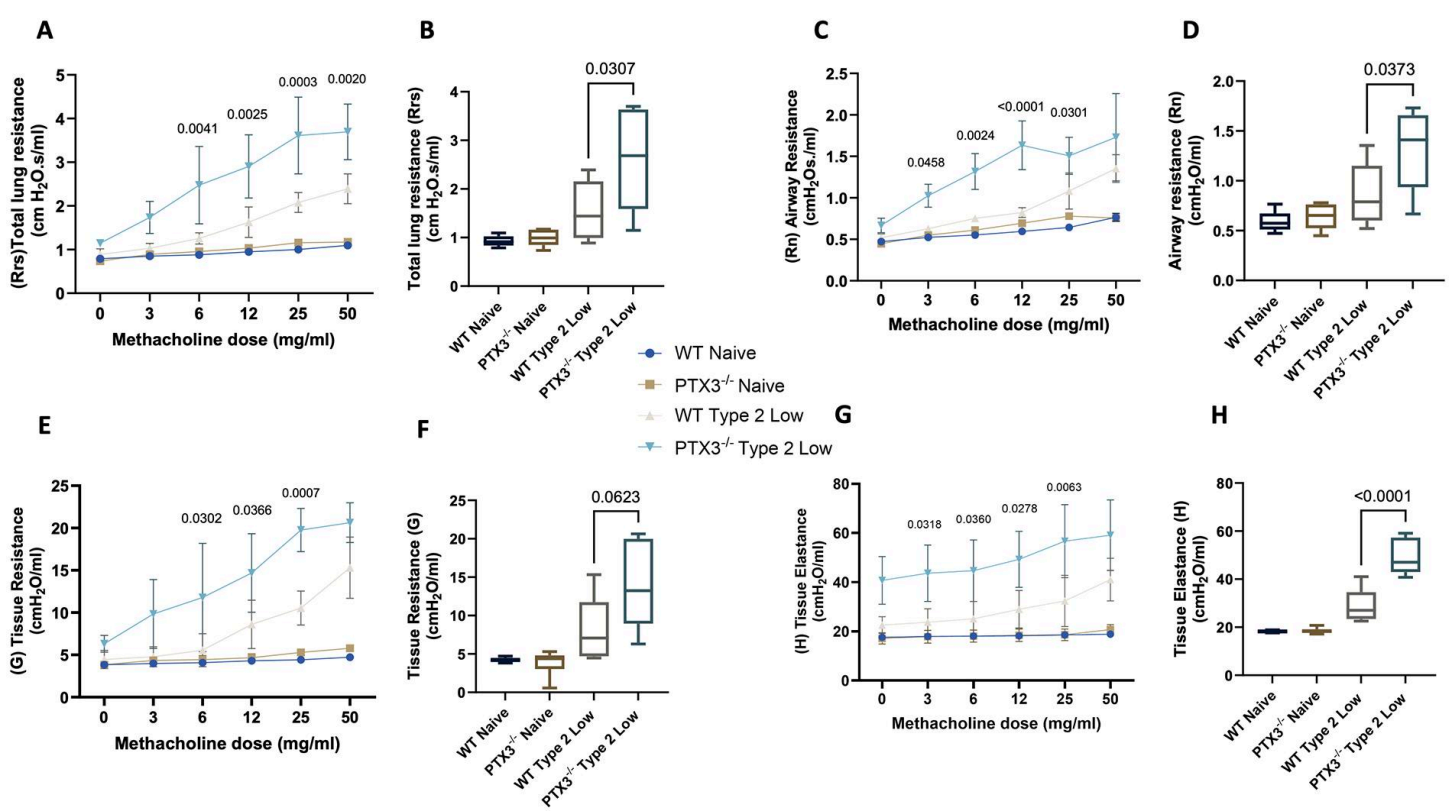
PTX3 deficiency increases airway and tissue resistance in the type 2-low asthma model. **(A)** Total lung resistance (Rrs) in response to increasing doses of methacholine. **(B)** Cumulative Rrs. **(C)** Airway resistance (Rn) across doses. **(D)** Cumulative Rn. **(E)** Tissue resistance **(G)** and **(F)** cumulative G. **(G)** Tissue elastance **(H)** and **(H)** cumulative H. Lung mechanics were measured using the FlexiVent system. (*n* = 8–10). Statistical analysis was performed using repeated-measures two-way ANOVA (dose–response curves) or one-way ANOVA (cumulative values). *P* values are reported on the figure.

PTX3^−/−^ type 2-low mice exhibited significantly elevated total lung resistance (Rrs) compared to WT type 2-low mice across multiple methacholine doses (Figure 5A), with cumulative Rrs also significantly increased (Figure 5B). Similarly, airway resistance (Rn) was significantly higher in PTX3^−/−^ mice than in WT controls, particularly at higher methacholine doses (Figure 5C), and this trend was reflected in the cumulative Rn values (Figure 5D).

PTX3^−/−^ type 2-low mice also showed significantly greater tissue resistance (G) at higher methacholine concentrations (Figure 5E), as well as higher cumulative G values (Figure 5F). Furthermore, tissue elastance (H) was markedly elevated in PTX3^−/−^ mice both across the dose range (Figure 5G) and in cumulative values (Figure 5H), suggesting impaired lung compliance.

These findings demonstrate that PTX3 deficiency is associated with significantly increased AHR in the type 2-low asthma model, with elevated airway and tissue resistance and stiffness in response to bronchoconstrictive stimuli.

## Discussion

Our findings shed light on the protective role of PTX3 in the severe, type 2-low asthma model and how its absence exacerbates key pathological features. In this study, PTX3 deficiency led to markedly shift in airway inflammation, characterized by dominant neutrophilic infiltration, a shift toward Th17 cytokine profiles, notably IL-17A, elevated IgE levels, and heightened airway AHR. These results align with emerging evidence that PTX3 acts as an endogenous regulator of inflammation in the lungs (22, 23).

Indeed, while PTX3 is typically upregulated during asthmatic inflammation as an acute-phase reactant and has been identified as a biomarker in asthma and other inflammatory diseases, its net effect in chronic airway disease appears to be anti-inflammatory and protective. Increased PTX3 levels have been associated with more severe asthma phenotypes characterized by neutrophil predominance and Th17-skewed responses (16).

Despite protective role of PTX3 in experimental model of type 2-low asthma, patients with severe asthma often exhibit elevated PTX3 levels without corresponding disease improvement (24). This paradox likely reflects a compensatory yet insufficient anti-inflammatory response. Severe asthmatics have intense neutrophilic inflammation and tissue damage (25) that triggers high PTX3 production (via pro-inflammatory cues like IL-1β and TNF*α*). However, the protective effect of PTX3 is overwhelmed by the magnitude and complexity of chronic severe asthma. In fact, PTX3 deficiency in mice selectively amplifies IL-17A-driven neutrophilic airway inflammation and steroid-resistant features, supporting the role of PTX3 in restraining severe type 2-low pathways. Yet, in established severe asthma, even abundant PTX3 cannot fully counteract these pathways, as the underlying Th17/neutrophil-dominated inflammation and corticosteroid-insensitivity persist unabated. More importantly, the role of PTX3 is context dependent (8, 26). While it normally helps resolve inflammation (e.g., by limiting IL-17 and neutrophil recruitment), sustained high PTX3 might also indicate ongoing innate immune activation rather than effective resolution. Indeed, exogenous PTX3 administration in allergic asthma models was shown to exacerbate airway eosinophilia, neutrophilia, and remodeling, underscoring a complex, double-edged function (27). It is also possible that qualitative differences in PTX3 underlie its lack of efficacy in severe asthma for instance, altered PTX3 isoforms or impaired PTX3 receptor interactions in severe disease could render the high levels functionally ineffectual (28). Evidence suggests that the oligomeric state of PTX3 may determine its biological activity. In other inflammatory conditions such as sepsis, persistently elevated levels of oxidized, octameric PTX3 were associated with poor clinical outcomes, whereas the presence of reduced or monomeric forms correlated with resolution and survival (28). This finding implies that high PTX3 in severe asthma may reflect the accumulation of structurally inactive or pro-inflammatory oligomers, rather than a protective form of the protein. Thus, total PTX3 levels may not accurately reflect functional anti-inflammatory capacity, and redox-regulated conformational changes could account for its failure to suppress inflammation in advanced disease. In addition, severe asthmatics often have steroid-unresponsive inflammation; since glucocorticoids can upregulate PTX3 in airway cells (29), a failure of this mechanism in severe disease may leave PTX3 elevation as a futile feedback loop rather than a curb on inflammation.

A striking outcome of PTX3 deficiency was the exaggerated neutrophilic inflammation in the lungs. PTX3 mice exhibited significantly higher neutrophil counts in bronchoalveolar lavage fluid and dense peribronchial neutrophil infiltration compared to WT controls. This mirrors clinical observations in severe asthma, where neutrophil-dominant airway inflammation is linked to frequent exacerbations, airflow obstruction, and poor corticosteroid responsiveness (30). Mechanistically, our results are consistent with the known function of PTX3 in restraining neutrophil recruitment. PTX3 can bind to the endothelial adhesion molecule P-selectin, thereby attenuating neutrophil extravasation at sites of inflammation (13). In the absence of PTX3, this braking mechanism is lost, contributing to unchecked neutrophil influx into the airways. Consequently, neutrophils likely release greater quantities of proteases (such as elastase) and reactive oxygen species, which can injure airway tissues, induce mucus hypersecretion, and promote remodeling (30). These effects synergistically contribute to the enhanced AHR observed in PTX3-deficient mice. Additionally, PTX3 plays a role in the resolution phase of inflammation by aiding the clearance of dying neutrophils. PTX3 can act as an “eat-me” signal or opsonin for apoptotic neutrophils, facilitating their uptake by macrophages (31). Thus, PTX3 deficiency may impair the efficient clearance of neutrophils, thereby prolonging the lifespan of inflammatory neutrophils in airway tissues and further perpetuating inflammation. Taken together, our data indicate that endogenous PTX3 serves as a critical negative regulator of neutrophilic inflammation in the asthmatic lung and its presence limits neutrophil recruitment and aids in the timely removal of spent neutrophils, whereas its absence unleashes persistent, tissue-damaging neutrophilia.

Coupled with neutrophilia, PTX3^−/−^ mice displayed significantly elevated IL-17A levels, suggesting activation of IL-17–associated inflammatory cells. While IL-17A is commonly associated with Th17 cells, it can also be produced by other immune populations, including γδ T cells and innate lymphoid cells 3 (ILC3) (32). Therefore, although our data demonstrate enhanced IL-17A, they do not definitively establish Th17 cells as the sole cellular source. This finding agrees with Balhara et al., who reported that PTX3 deletion in an OVA asthma model promotes a Th17-dominant phenotype, accompanied by enhanced neutrophilia (16). IL-17A is a potent neutrophil-recruiting cytokine and a known driver of steroid-resistant asthma pathology (33). The elevated IL-17A in PTX3 deficient airways likely plays a central role in amplifying neutrophilic inflammation, for example, by inducing CXCL chemokines that attract neutrophils, thereby creating a self-reinforcing cycle of inflammation. In support of this, we and others have found that PTX3^−/−^ dendritic cells produce higher amounts of IL-6 and IL-23, which are key cytokines for Th17 differentiation and maintenance. The excess of these Th17-polarizing signals in PTX3 deficiency provides a fertile environment for IL-17 producing CD4^+^ T cells to thrive (16, 23). It is also important to consider innate sources of IL-17A in this context. γδ T cells and certain innate lymphoid cells (34) can produce IL-17A and have been implicated in neutrophilic airway inflammation, especially in severe asthma and experimental models of chronic lung disease (35–37). PTX3 may normally restrain these innate IL-17 sources; for instance, exogenous PTX3 has been shown to suppress IL-17 mediated immunopathology by limiting γδ T cell expansion in models of chronic infection and inflammation (38–40). Altogether, the Th17 bias in PTX3 KO mice provides a plausible mechanistic link between the absence of PTX3 and the steroid-insensitive, severe asthma phenotype, since Th17 pathways are strongly associated with corticosteroid resistance and refractory asthma exacerbations. Our data reinforce the notion that PTX3 is a critical brake on Th17-driven inflammation in the airways, and losing this brake skews the immune response toward a more severe neutrophilic profile.

In addition to cellular inflammation, PTX3 deficiency had notable effects on humoral immune responses. We observed significantly elevated total and HDM-specific IgE levels in PTX3^−/−^ mice compared to WT, indicating an exaggerated allergic antibody response in the absence of PTX3. Notably, although the type 2-low asthma model used in this study is characterized predominantly by neutrophilic inflammation, it also induces modest eosinophilia, reflecting a mixed granulocytic inflammatory environment. Importantly, both WT and PTX3^−/−^ mice were exposed to identical sensitization and challenge conditions, and eosinophil levels within the type 2-low model were comparable between genotypes, indicating that the selective increase in IgE observed in PTX3^−/−^ mice is not solely attributable to residual type 2 inflammation. This finding is in line with previous work demonstrating heightened IgE in PTX3 KO conditions (16). At first glance, the increase in IgE might seem paradoxical given that Th2 cytokines (classically required for IgE class-switching) were not elevated in the PTX3^−/−^ mice. However, PTX3 might play a role during the initial sensitization to allergens. Its absence could lead to an unrestrained early Th2 activation, with even transient IL-4 production being sufficient to drive B cells toward IgE. Balhara et al. noted that PTX3 KO CD4^+^ T cells have reduced IL-2 and enhanced survival/activation after allergen exposure (16). Diminished IL-2 (a T cell regulatory cytokine) and prolonged T cell survival could allow sustained interactions between T helper cells and B cells, facilitating ongoing IgE class switching even if peak IL-4 levels are lower. In essence, a more persistent T cell help, biased by the PTX3 deficient milieu, may compensate for the quantity of IL-4 by extending the duration of B cell activation. The excess IL-6 in PTX3^−/−^ mice might indirectly promote IgE. IL-6 can contribute to alternative B-cell help through T follicular helper cells and IL-21 and has been implicated in enhancing antibody production in chronic inflammation (41). Moreover, IL-6 together with IL-1β and IL-23 drives Th17 differentiation; Th17 cells can co-exist with Th2 responses and have been reported to assist B cells in certain contexts (42). The net cytokine milieu in PTX3 deficiency, high IL-6, IL-17A, and possibly higher IL-13 and TGF-β could synergistically favor IgE production and airway remodeling.

Functionally, the inflammatory changes in PTX3 KO mice translated into worse lung mechanics. Consistent with prior reports (16), we found that airway hyperresponsiveness to methacholine was significantly augmented in PTX3^−/−^ animals relative to WT. This is an expected consequence of the heightened inflammation: neutrophils and IL-17A can both contribute to AHR through multiple pathways. Neutrophil-derived products, such as elastase, have been shown to directly induce bronchoconstriction, goblet cell hyperplasia, and airway smooth muscle hypercontractility, thereby increasing AHR (43–45).

Although the use of a global PTX3 knockout model allows mechanistic inference regarding PTX3 function in airway inflammation, it also carries inherent limitations. Developmental compensation and systemic effects of lifelong PTX3 deficiency cannot be fully excluded. In addition, while the HDM + c-di-GMP protocol reproduces key features of human type 2-low asthma, including neutrophilic inflammation and IL-17A elevation, it remains a preclinical model and does not capture the full heterogeneity of the human disease.

In summary, the absence of PTX3 profoundly worsens the course of experimental asthma, highlighting PTX3 as an essential negative regulator of airway inflammation. PTX3 deficiency skews the immune response toward a neutrophil and IL-17A rich axis, with accompanying increases in IgE and AHR, thereby recapitulating features of the most severe forms of asthma. These results not only deepen our understanding of asthma endotypes illuminating why some patients with low PTX3 activity may develop neutrophilic, therapy-resistant disease but also point toward PTX3-centric pathways as potential therapeutic targets. Augmenting the function of PTX3 in the lung, or mimicking its regulatory effects, could emerge as a novel strategy to alleviate neutrophilic airway inflammation and restore balance in the asthmatic immune response. Further investigations are warranted to dissect the precise molecular interactions of PTX3 in the asthmatic lung and to determine how we might safely harness its protective powers to benefit patients with severe asthma.

## Data Availability

The datasets presented in this study can be found in online repositories. The names of the repository/repositories and accession number(s) can be found in the article/Supplementary Material.
