# Supplementary material for: Pentraxin 3 deficiency exacerbates neutrophilic inflammation and airway hyperresponsiveness in type 2-low asthma
Source: Front Allergy. 2026 Feb 5;7:1731295. doi: 10.3389/falgy.2026.1731295 (PMC12916562; doi:10.3389/falgy.2026.1731295)
Supplement: Supplementary file 1 [file Table1.docx]

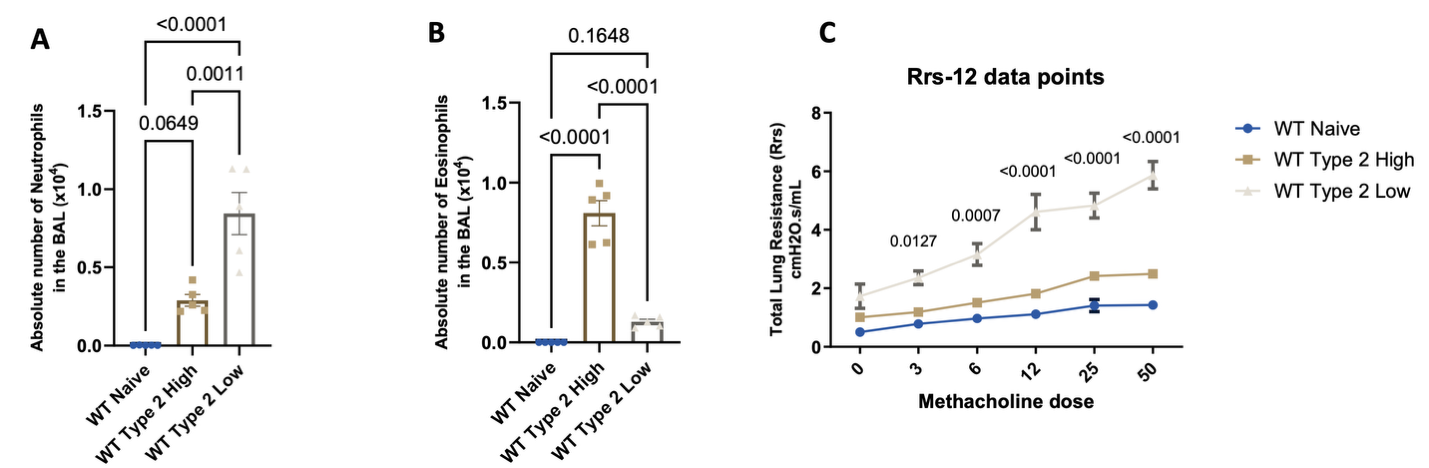


**Figure 1S. Characterization of airway inflammation in chronic murine models of type 2-low and type 2-high asthma.** (A) Absolute numbers of neutrophils in BALF determined by flow cytometry across naïve, type 2-high, and type 2-low groups. (B) Absolute numbers of eosinophils in BALF determined by flow cytometry in the same groups. Each dot represents one individual biological replicate. Statistical analysis was performed using one-way ANOVA or repeated-measures two-way ANOVA, as appropriate.
